# Supplementary material for: Saliva TwoStep for rapid detection of asymptomatic SARS-CoV-2 carriers
Source: eLife. 2021 Mar 29;10:e65113. doi: 10.7554/eLife.65113 (PMC8057811; doi:10.7554/eLife.65113)
Supplement: Peer review history

# Decision letter

**DOI:** 10.7554/eLife.65113.sa1

**Affiliations:** University of the WitwatersrandSouth Africa; Heidelberg University HospitalGermany

In the interests of transparency, eLife publishes the most substantive revision requests and the accompanying author responses.

**Acceptance summary:**

This study provides a novel strategy for the diagnosis of SARS-CoV-2 infection using a simple detection system for viral genomic material, together with saliva as the clinical specimen. The authors showed that acidic saliva resulted in false-positive results, and to avoid this false positivity, NaOH was added to saliva samples. The utility of this strategy was tested using clinical saliva samples, demonstrating high sensitivity and specificity. The work will be of interest to clinical microbiologists and virologists.

**Decision letter after peer review:**

Thank you for submitting your article "Saliva TwoStep for rapid identification of SARS-CoV-2 carriers in a large population" for consideration by *eLife*. Your article has been reviewed by 3 peer reviewers, and the evaluation has been overseen by Bavesh Kana as the Senior and Reviewing Editor. The following individuals involved in review of your submission have agreed to reveal their identity: Petr Chlanda (Reviewer #2); Viet Loan Dao Thi (Reviewer #3, co-reviewed with Prof. Michael Knop).

The reviewers have discussed the reviews with one another and the Reviewing Editor has drafted this decision to help you prepare a revised submission.

As the editors have judged that your manuscript is of interest, but as described below that additional experiments are required before it is published, we would like to draw your attention to changes in our revision policy that we have made in response to COVID-19 (https://elifesciences.org/articles/57162). First, because many researchers have temporarily lost access to the labs, we will give authors as much time as they need to submit revised manuscripts. We are also offering, if you choose, to post the manuscript to bioRxiv (if it is not already there) along with this decision letter and a formal designation that the manuscript is "in revision at *eLife*". Please let us know if you would like to pursue this option. (If your work is more suitable for medRxiv, you will need to post the preprint yourself, as the mechanisms for us to do so are still in development.)

Summary:

The authors develop a diagnostic testing approach for detecting SARS-CoV-2 in saliva using loop-mediated isothermal amplification (LAMP) and sodium hydroxide to adjust the pH of samples. They tested the utility of the approach using clinical saliva samples (both PCR negative and PCR positive samples) thus demonstrating high sensitivity and specificity of their test. As SARS-CoV-2 infections increase around the world, this approach promises to deliver public health benefit.

Key findings:

1. The authors used NaOH to adjust the pH of the saliva before RT-LAMP and found that 14.5 mM NaOH is sufficient to neutralize the saliva without compromising RT-LAMP detection.

2. The method was validated on a cohort of human saliva samples (295 negatives, 168 positives) that were tested by RT-PCR.

The presented work provides a promising step towards rapid and inexpensive testing for SARS-CoV-2.

Essential revisions:

1. An important matter of concern is the sensitivity of the assay. The authors state that they investigated this using samples from university testing. This university testing was conducted using a direct RT-qPCR assay with a limit of detection (LOD) of approx. 5000 viral copies per ml of saliva sample. This reference (their ref 12) should be included also on page 25. This LOD is approx. 10x lower (3-4 CT steps) than RT-qPCR based on isolated RNA and using commercial state of the art RT-qPCR kits. The best commercial RT-qPCR kits, which employ novel primer designs and semi-nested PCR strategies that can detect a single RNA molecule in the reaction with >95% sensitivity, are roughly 50x (5-6 CT steps) more sensitive than the direct RT-qPCR assay used in this study. The authors explained that they used from this student cohort "295 negative samples, and 168 positive samples with viral loads at or above our limit of detection (200 virions/μL)". For this testing, they used 1 μl of saliva volume which corresponds to a sensitivity of 200'000 genomes per ml of saliva. From this, several questions arise: The 168 positive samples tested: from how many different people do they origin? Are there also longitudinal sample series included? What fraction of the RT-qPCR positive samples of the university testing contained a lower viral load than the detection limit estimate from Figure S4, and hence were not considered for the analysis? Why was the analysis limited to this high-load sample range? If they had included also the remaining positive samples, they could have better calculated the sensitivity as a function of the CT of diagnostic RT-qPCR. Samples with low viral loads must be included in the revision. From this, the authors can calculate the fraction of students they would have missed when using RT-LAMP instead of RT-qPCR, and, when combined with contact tracing data (which they seem to have available), they could have tried to estimate the impact of their RT-LAMP procedure vs the used RT-qPCR strategy. What would be the LOD if the results of both primer sets were combined?

2. No data are shown on the prevalence of acidic saliva in the tested cohort. It is not known how many samples were acidic, and it could also be that all tested negative samples had a neutral pH. Further, no data is available on whether the addition of NaOH to already neutral saliva will decrease the detection limit. The study could be improved by providing RT-LAMP data performed with and without NaOH on the same set of samples. This could be done by using RT-LAMP with and without NaOH on the same set of samples and show photographs of the test tubes before and after heat incubation. Besides, this would also allow direct determination of the false positive number in cases where NaOH is not used. Related to this, please provide data that would support your statement on page 4, "In fact, we find about 10% of human saliva samples are naturally acidic enough to immediately trigger phenol red-containing reactions to change to yellow without any target amplification required." The number of naturally acidic saliva samples in the cohort is not reported.

3. Figure 1. Samples with 0 mM NaOH are missing to compare the colour change. An addition of 14.5 mM of NaOH to all samples might decrease the detection limit of RT-LAMP. In particularly SARS-CoV-2 positive saliva samples that are naturally more alkaline than others and have a low viral load might be missed because of the addition of NaOH. Figure 3 shows that the detection limit of RT-LAMP is around 200 virions/µl (Ct ~ 30) in the presence of 14.5 mM NaOH. The detection limit, specificity, and sensitivity for naturally neutral saliva with and without the addition of 14.5mM NaOH should be determined and directly compared, using both high and low viral load samples.

4. Page 18: Please provide data that the stabilization solution helps to preserve saliva samples for at least four days. Supplementary Figure S2 shows that saliva samples were spiked with heat-inactivated SARS-CoV-2 virions and mixed 1:1 with the stabilization solution but no dataset is provided without the stabilizing solution.

[Editors' note: further revisions were suggested prior to acceptance, as described below.]

Thank you for submitting your article "Saliva TwoStep for rapid detection of asymptomatic SARS-CoV-2 carriers" for consideration by *eLife*. Your article has been reviewed by 2 peer reviewers, and the evaluation has been overseen by Bavesh Kana as the Senior and Reviewing Editor. The following individuals involved in review of your submission have agreed to reveal their identity: Petr Chlanda (Reviewer #2); Viet Loan Dao Thi (Reviewer #3).

The reviewers have discussed their reviews with one another, and the Reviewing Editor has drafted this to help you prepare a revised submission. Reviewers concur that your manuscript is improved but there are still some outstanding issues that require further clarity. These are detailed below.

Essential Revisions:

1. For comparison of RT-LAMP and RT-PCR, you included in the first manuscript only samples with a CT<30. The revised selection of samples with a CT>30 are distributed in a non-natural bimodal fashion. This indicates that the samples are not from a coherent dataset, either from different datasets with different PCR methods, or there must have been some sort of pre-selection of samples. If you have preselected or filtered these specimens, they are not suitable to calculate sensitivity. Please explain the source of the samples/data and that the analysis in 4B and 4C is omitted.

2. Where it states, "…the usage of dUTP and uracil-DNA-glycosylase-containing RT-LAMP reaction mix can be considered. Through data not shown, we determined that the addition of this alternative master mix does not affect the reported test limit of detection." This data should be shown or the statement about the mix should be omitted.

3. Please explain who you would interpret positive RT-LAMP, negative RT-qPCR

(as a comparator)? If the sensitivity of your assay is much lower than a direct RT-PCR assay and no other advantage is provided, why should one use RT-LAMP for testing at all?

Reviewer #2:

Qing Yang et al. have addressed all my comments and provided a substantial amount of new data to show that stabilizing solution is improving RT-LAMP performance on saliva samples without an RNA purification step. They show that the addition of NaOH is not decreasing the sensitivity of the assay. The authors provide improved figures and nicely show that the addition of stabilizing solution shortly after collection increases the stability of saliva samples for at least 4 days. They also directly assayed the prevalence of naturally acidic saliva and show that it is about 7%, which clearly justifies the importance of their work. I congratulate the authors on their work and recommend it for publication without additional revisions.

Reviewer #3:

The present manuscript is an improved version by Yang et al., who describe the development of a rapid molecular test for SARS-CoV-2, based on RT-LAMP detection in saliva samples.

For comparison of RT-LAMP and RT-PCR, the authors included in the first manuscript only samples with a CT<30. Now they include also samples with a CT>30, but the values are distributed in a non-natural bimodal fashion (Figure 4A9#). This indicates that the samples are not from a coherent dataset, either from different datasets with different PCR methods, or there must have been some sort of pre-selection of samples. This simply means that the data is not suitable to calculate sensitivity at all! – We suggest that the authors explain better the source of the samples/data and that the analysis in 4B and 4C is omitted.

The following point should be still addressed: "…the usage of dUTP and uracil-DNA-glycosylase-containing RT-LAMP reaction mix can be considered. Through data not shown, we determined that the addition of this alternative master mix does not affect the reported test limit of detection." This data should be shown or the statement about the mix should be omitted.

Point 8. The authors seem to confirm the idea that the used Lyra PCR assay is of limited sensitivity. Since PCR is still considered to be the gold standard, we would like to reiterate our question: If the authors would use the RT-LAMP assay for diagnostics (and not only to re-assay samples that have already been assayed): how would they deal with such a situation – positive RT-LAMP, negative RT-qPCR? What diagnostic result is given out to the participant? This is an important question that has legal implications.

Point 9: If the sensitivity of their assay is much lower than a direct RT-PCR assay and no other advantage is provided, why should one use RT-LAMP for testing at all?

Other than these points, the authors have addressed all other questions.

Few comments that could, but not necessarily, be addressed:

Line 190: This suggests the observed detection limit represents the upper performance limit of colorimetric RT-LAMP, and the saliva and stabilization solution have little to no negative impact to the test performance"

Other studies have shown that the detection limit of the colorimetric RT-LAMP assay is 50-100 genome copies/ul. Hence, I would re-phrase this sentence.

Line 220: "Because positive results in our university screening regimen result in university affiliates being directed to their healthcare provider for confirmatory testing, with a few exceptions every positive sample is from a unique individual."

Does that mean that the positively tested candidates were removed from the participant's pool, which is why we can assume that each sample corresponds to one individual? This could be rephrased to make it more clear.

# Reply: Author response

**DOI:** 10.7554/eLife.65113.sa2

Essential revisions:

1. An important matter of concern is the sensitivity of the assay. The authors state that they investigated this using samples from university testing. This university testing was conducted using a direct RT-qPCR assay with a limit of detection (LOD) of approx. 5000 viral copies per ml of saliva sample. This reference (their ref 12) should be included also on page 25. This LOD is approx. 10x lower (3-4 CT steps) than RT-qPCR based on isolated RNA and using commercial state of the art RT-qPCR kits. The best commercial RT-qPCR kits, which employ novel primer designs and semi-nested PCR strategies that can detect a single RNA molecule in the reaction with >95% sensitivity, are roughly 50x (5-6 CT steps) more sensitive than the direct RT-qPCR assay used in this study.

We have now added significantly more detail to the methods section regarding the methods used in university testing, including adding the reference but also additional details as well.

The reviewer is correct that the detection limit of RT-qPCR is different depending on whether the template is purified RNA or saliva. We have been more careful to discuss this difference and to state the LoDs of each, where appropriate.

The authors explained that they used from this student cohort "295 negative samples, and 168 positive samples with viral loads at or above our limit of detection (200 virions/μL)". For this testing, they used 1 μl of saliva volume which corresponds to a sensitivity of 200'000 genomes per ml of saliva. From this, several questions arise: The 168 positive samples tested: from how many different people do they origin? Are there also longitudinal sample series included?

This is a good point which we now clarify. We have added the following text to the beginning of the Results section where we describe out sample cohort: “Because positive results in our screening regimen result in university affiliates being directed to their healthcare provider, with a few exceptions every positive sample is from a unique individual. “

What fraction of the RT-qPCR positive samples of the university testing contained a lower viral load than the detection limit estimate from Figure S4, and hence were not considered for the analysis? Why was the analysis limited to this high-load sample range? If they had included also the remaining positive samples, they could have better calculated the sensitivity as a function of the CT of diagnostic RT-qPCR. Samples with low viral loads must be included in the revision. From this, the authors can calculate the fraction of students they would have missed when using RT-LAMP instead of RT-qPCR, and, when combined with contact tracing data (which they seem to have available), they could have tried to estimate the impact of their RT-LAMP procedure vs the used RT-qPCR strategy.

Done! We have now included the data requested in Figure 4A. As suggested, we also added the panel new Figure 4B, which illustrates the RT-LAMP sensitivity as a function of the RT-qPCR Ct values. In addition, we calculated the test sensitivity considering the entire cohort (new Figure 4C).

What would be the LOD if the results of both primer sets were combined?

We did such an experiment (Figure 3—figure supplement 2). When a test is considered positive only if both of the primer sets turn positive, the observed LOD using the positive saliva samples is 1607 virions/μl. If a test is considered positive if either of the primer sets turns positive, the observed LOD is 102 virions/μl. As would be expected, it lowers the sensitivity a little when you require two non-perfect primer pairs to both agree.

2. No data are shown on the prevalence of acidic saliva in the tested cohort. It is not known how many samples were acidic, and it could also be that all tested negative samples had a neutral pH. Further, no data is available on whether the addition of NaOH to already neutral saliva will decrease the detection limit. The study could be improved by providing RT-LAMP data performed with and without NaOH on the same set of samples. This could be done by using RT-LAMP with and without NaOH on the same set of samples and show photographs of the test tubes before and after heat incubation. Besides, this would also allow direct determination of the false positive number in cases where NaOH is not used. Related to this, please provide data that would support your statement on page 4, "In fact, we find about 10% of human saliva samples are naturally acidic enough to immediately trigger phenol red-containing reactions to change to yellow without any target amplification required." The number of naturally acidic saliva samples in the cohort is not reported.

We did all of these experiments and added them to the paper.

To test for the prevalence of the naturally acidic saliva, we tested 96 saliva samples and examined how many would turn the RT-LAMP reaction with or without the optimized stabilization solution (New Panel A in Figure 1).

We also did the requested experiments demonstrating that the stabilization solution helps prevent false positivity prior to incubation for RT-LAMP amplification, without creating false negatives. The experiments that the reviewers suggested are now shown in new Figure 3—figure supplement 1.

3. Figure 1. Samples with 0 mM NaOH are missing to compare the colour change.

It is a reasonable request but actually quite difficult to do that control (although we did it and show the new data in Author response image 1). Because it contains TCEP-HCl, the stabilization solution is acidic without the addition of any NaOH. This causes all RT-LAMP reactions turned yellow even before incubation for RT-LAMP amplification, just as acidic saliva samples also do.

An alternative negative control for 0 mM NaOH would be adding raw saliva directly into the RTLAMP reaction mix without any saliva stabilization solution at all. We have now done this experiment and it is shown in the new Figure 3—figure supplement 1 (panel B).

**Author response image 1.**

An addition of 14.5 mM of NaOH to all samples might decrease the detection limit of RT-LAMP. In particularly SARS-CoV-2 positive saliva samples that are naturally more alkaline than others and have a low viral load might be missed because of the addition of NaOH. Figure 3 shows that the detection limit of RT-LAMP is around 200 virions/µl (Ct ~ 30) in the presence of 14.5 mM NaOH. The detection limit, specificity, and sensitivity for naturally neutral saliva with and without the addition of 14.5mM NaOH should be determined and directly compared, using both high and low viral load samples.

In response to editors’ comments, we evaluated the RT-LAMP detection limit in the presence and absence of stabilization solution. First, we measured the RT-LAMP detection upper limit using synthetic SARS-CoV-2 RNA diluted in nuclease-free water and directly added to the RTLAMP master mix (new Figure 3—figure supplement 1, panel A). It is 200 genome copies/µl. We then confirmed that such detection limit remains at 200 virions/µl when using pH-neutral saliva spiked-in with heat-inactivated SARS-CoV-2 virions and mixed 1:1 with stabilization solution containing 14.5 mM NaOH (new Figure 3—figure supplement 1, panel B). This suggests neither the saliva nor the stabilization solution has negative effect on the RT-LAMP detection limit.

To more directly address the question of whether the NaOH-containing stabilization solution has negative impact on RT-LAMP detection limit, we also attempted mixing pH-neutral saliva spiked-in with virions 1:1 with nuclease-free water. The samples were again heated at 95ºC for 10 minutes before added to RT-LAMP reaction mix. However, we observed inconsistent results (or detection limit > 800 virions/µl), in the absence of stabilization solution (new Figure 3—figure supplement 1, panel B). This is likely due to RNA degradation due to heat and/or nuclease.

4. Page 18: Please provide data that the stabilization solution helps to preserve saliva samples for at least four days. Supplementary Figure S2 shows that saliva samples were spiked with heat-inactivated SARS-CoV-2 virions and mixed 1:1 with the stabilization solution but no dataset is provided without the stabilizing solution.

We have now added extensive control conditions including those requested. We re-did the experiment described in now Figure 1—figure supplement 2 with the additional control conditions and remade a new Figure 1—figure supplement 2. Due to the rather complex experimental setup, we included panel A in Figure 1—figure supplement 2 illustrating the experimental conditions. Based on the results, we observed that the conditions used in our test (condition C in the new figure) were able to preserve the RNA in the samples and maintain the detection limit of 200 virions/µl for at least 96 hours. However, both control conditions failed to preserve human and viral RNA, thus resulting in inconsistent results.

[Editors' note: further revisions were suggested prior to acceptance, as described below.]

Essential Revisions:

1. For comparison of RT-LAMP and RT-PCR, you included in the first manuscript only samples with a CT<30. The revised selection of samples with a CT>30 are distributed in a non-natural bimodal fashion. This indicates that the samples are not from a coherent dataset, either from different datasets with different PCR methods, or there must have been some sort of pre-selection of samples. If you have preselected or filtered these specimens, they are not suitable to calculate sensitivity. Please explain the source of the samples/data and that the analysis in 4B and 4C is omitted.

We understand the concern perfectly and have triple-checked everything. The data are all from one sample set, and no pre-screening was done. SARS-CoV-2 positive saliva samples were collected from September 16 – September 25, 2020, from which *all* positive saliva samples above the RT-qPCR detection limit were used for RT-LAMP validation illustrated in Figure 4A. The RT-qPCR method used for the SARS-CoV-2 screening is consistent during this time period, with the details described in the existing method section.

The “non-natural bimodal” observation by the reviewer is a combined result of two things: (1) The large majority of SARS-CoV-2 positive samples had a Ct value for N gene centered around 29-32; and (2) the separation of RTLAMP positive and negative samples from the same population for clear visualization. In addition, we also noticed the lack of separation of the data points in Figure 4A could lead to visual confusion, and subsequently fixed the figure width to address that problem.

To clarify this matter further, we added a figure showing the overall SARS-CoV-2 N gene

Ct distribution of all samples collected during the stated time period (Figure 4—figure supplement 2A). In Figure 4A, We validated RT-LAMP TwoStep assay using all samples with N gene Ct value lower than the RT-qPCR detection limit (Ct<34, or 5 virions/µL, highlighted in dark grey in Figure 4—figure supplement 2A).

Nevertheless, the overall sampling is unbiased, and the N gene Ct value distribution is near normal, with outliers only near the extreme values (Figure 4—figure supplement 2B, D'Agostino test, K^2^=9.07, p-value=0.011).

We increased the width of Figure 4A to avoid data points overlapping each other.

We included Figure 4—figure supplement 2 to illustrate the overall distribution of the SARS-CoV-2 N gene Ct values of all SARS-CoV-2 positive saliva samples used for RT-LAMP validation.

We included Appendix 2, which summarizes RT-qPCR results and the corresponding RT-LAMP results for all the university samples tested.

2. Where it states, "…the usage of dUTP and uracil-DNA-glycosylase-containing RT-LAMP reaction mix can be considered. Through data not shown, we determined that the addition of this alternative master mix does not affect the reported test limit of detection." This data should be shown or the statement about the mix should be omitted.

The comparison of the RT-LAMP master mix with or without dUTP and UDG was done using limited reagents (the dUTP and UDG RT-LAMP master mix was offered to us in trial size), so the supporting experiment lacked sufficient replicates. Instead, we rephrased the statement in the manuscript, and cited relevant published articles.

Instead of the original statement, “through data not shown, we determined that the addition of this alternative master mix does not affect the reported test limit of detection”, we rephrased this sentence in the Discussion section with relevant references: DOI: 10.1101/2020.06.23.166397 and DOI: 10.1038/srep27605

3. Please explain who you would interpret positive RT-LAMP, negative RT-qPCR

(as a comparator)? If the sensitivity of your assay is much lower than a direct RT-PCR assay and no other advantage is provided, why should one use RT-LAMP for testing at all?

The RT-LAMP TwoStep test described herein is aimed to be used as a community screening test. In a typical screening setting, an individual who receives a positive screening test (RT-LAMP) result will be directed to get a confirmatory diagnostic test (RT-qPCR in CLIA lab).

Given that no false-positive was observed during our validation, in the event when RTLAMP is positive while the follow-up RT-qPCR is negative, the discrepancy might be explained by the differences in the timing of sample collection and/or the specimen types. It will be up to physicians and policy makers to decide whether subsequent quarantine is needed. However, such subject would be out of the scope of this research paper.

On the other hand, as stated in the Discussion section, we highlighted the advantages of RT-LAMP in comparison to RT-qPCR: “Saliva TwoStep requires less sample processing, reaction incubation time, and laboratory overhead as compared to quantitative RT-PCR methods. The result is the ability to run significantly more tests with a given amount of resources.”

Therefore, the use of screening test will allow an increased testing capacity within the asymptomatic community, and identification of SARS-CoV-2 positive individuals in remote or less-developed communities where equipment or RT-qPCR is less accessible.

Reviewer #3:

The present manuscript is an improved version by Yang et al., who describe the development of a rapid molecular test for SARS-CoV-2, based on RT-LAMP detection in saliva samples.

For comparison of RT-LAMP and RT-PCR, the authors included in the first manuscript only samples with a CT<30. Now they include also samples with a CT>30, but the values are distributed in a non-natural bimodal fashion (Figure 4A9#). This indicates that the samples are not from a coherent dataset, either from different datasets with different PCR methods, or there must have been some sort of pre-selection of samples. This simply means that the data is not suitable to calculate sensitivity at all! – We suggest that the authors explain better the source of the samples/data and that the analysis in 4B and 4C is omitted.

The following point should be still addressed: "…the usage of dUTP and uracil-DNA-glycosylase-containing RT-LAMP reaction mix can be considered. Through data not shown, we determined that the addition of this alternative master mix does not affect the reported test limit of detection." This data should be shown or the statement about the mix should be omitted.

Point 8. The authors seem to confirm the idea that the used Lyra PCR assay is of limited sensitivity. Since PCR is still considered to be the gold standard, we would like to reiterate our question: If the authors would use the RT-LAMP assay for diagnostics (and not only to re-assay samples that have already been assayed): how would they deal with such a situation – positive RT-LAMP, negative RT-qPCR? What diagnostic result is given out to the participant? This is an important question that has legal implications.

Point 9: If the sensitivity of their assay is much lower than a direct RT-PCR assay and no other advantage is provided, why should one use RT-LAMP for testing at all?

Other than these points, the authors have addressed all other questions.

Few comments that could, but not necessarily, be addressed:

Line 190: This suggests the observed detection limit represents the upper performance limit of colorimetric RT-LAMP, and the saliva and stabilization solution have little to no negative impact to the test performance"

Other studies have shown that the detection limit of the colorimetric RT-LAMP assay is 50-100 genome copies/ul. Hence, I would re-phrase this sentence.

We are aware of the reported RT-LAMP assay detection limit of 50-100 genome copies/uL by other studies. Our estimate on the detection limit is more conservative, basing on the evaluation using the large cohort of actual human samples. Nevertheless, we rephrase the sentence in the manuscript to be more accurate:

“This suggests the observed detection limit represents the upper performance limit of the presented RT-LAMP assay, and the saliva and stabilization solution have little to no negative impact to the test performance”

Line 220: "Because positive results in our university screening regimen result in university affiliates being directed to their healthcare provider for confirmatory testing, with a few exceptions every positive sample is from a unique individual."

Does that mean that the positively tested candidates were removed from the participant's pool, which is why we can assume that each sample corresponds to one individual? This could be rephrased to make it more clear.

We rephrased this sentence based on reviewers recommendation to add more clarity:

“Because positive results in our university screening regimen result in the tested individual being directed to their healthcare provider for confirmatory testing, positively tested individuals were removed from the sampling pool. Thus, most positive samples are from unique individuals, with a few exceptions.”
